# Next-generation sequencing-based molecular diagnosis of 12 inherited retinal disease probands of Uyghur ethnicity

**DOI:** 10.1038/srep21384

**Published:** 2016-02-09

**Authors:** Abulikemu Tajiguli, Mingchu Xu, Qing Fu, Rouzimaimaiti Yiming, Keqing Wang, Yumei Li, Aiden Eblimit, Ruifang Sui, Rui Chen, Haji Akber Aisa

**Affiliations:** 1 The Key Laboratory of Plant Resources and Chemistry of Arid Zone, Xinjiang Technical Institute of Physics and Chemistry, Chinese Academy of Sciences, Urumqi, Xinjiang, China 830011; 2 Department of Molecular and Human Genetics, Baylor College of Medicine, Houston, Texas, United States 77030; 3 Human Genome Sequencing Center, Baylor College of Medicine, Houston, Texas, United States 77030; 4 Department of Ophthalmology, North Huashan Hospital, Fudan University, Shanghai, China 200240; 5 Department of Ophthalmology, Minguang Ophthalmic Hospital, Hotan, Xinjiang, China 848000; 6 Department of Ophthalmology, Peking Union Medical College Hospital, Peking Union Medical College, Chinese Academy of Medical Sciences, Beijing, China 100730

## Abstract

Inherited retinal disease (IRD) is a category of genetic disorders affecting retina. Understanding the molecular basis of IRD is vital for clinical and genetic classification of patients. Uyghur people is an isolated ethnic group mainly residing in northwestern China with genetic admixture from Europeans and East Asians. The genetic etiology of IRD in this specific population still remains unknown. Here, by next-generation sequencing (NGS), we screened mutations in over 200 known retinal disease genes in a cohort of 12 unrelated Uyghur IRD probands. Out of the 12 probands, six are solved with high confidence, two with low confidence, while the remaining four are unsolved. We identified known disease-causing alleles in this cohort that suggest ancient Uyghur migration and also discovered eight novel disease-associated variants. Our results showed NGS-based mutation screening as a reliable approach for molecular diagnosis. In addition, this approach can also be applied to reveal the genetic history of a specific ethnic group.

Retina is the structure surrounding the back of the vitreous body in the eye. It transforms light information into electrophysiological signals for human visual perceptions. The retina consists of various types of cells with highly specialized structures for its specific functions. Therefore, a number of pathways play important roles in the survival and functions of retina, including photo-transduction^1^, ciliogenesis^2^,^3^, metabolism^4^, transcription regulation^5^,^6^, vascular development^7^, protein folding^8^, etc. Correspondingly, mutations in genes involved in these pathways will result in a series of genetic disorders named inherited retinal disease (IRD), including retinitis pigmentosa (RP, MIM# 268000), Leber congenital amaurosis (LCA, MIM# 204000), cone-rod dystrophy (CRD, MIM# 120970), familial exudative vitreoretinopathy (FEVR, MIM# 133780), etc. IRD shows highly complex genetic etiology, with diverse inheritance patterns and more than two hundred disease-causing genes identified^9^.

With the advent of next-generation sequencing (NGS), numerous studies have applied NGS technology to reveal the molecular basis of human Mendelian disease including IRD. Specifically, customized target capture sequencing was used to screen mutations in known disease-causing genes with high efficiency^7^,^10^,^11^. By this method, novel disease-causing alleles as well as genotype-phenotype correlations have been identified, thus greatly enhancing our understanding of allele pathogenicity, protein function and population genetics^7^,^10^,^11^. Hence, NGS-based molecular diagnosis has been proven as a robust approach for assessing Mendelian disease in a molecular level.

Uyghur people are a Turkic language-speaking ethnic group mainly residing in Tarim Basin of northwestern China. Tarim Basin is an extremely isolated region due to the semi-arid or desert climate and its bordering rugged Karakoram, Kunlun and Tianshan mountain ranges. The Uyghur people were greatly influenced by Middle East Muslim culture after 10^th^ century A.D. and they possess distinct genetic admixture derived from both East Asians and Europeans^12^. Previous genetic studies in Uyghur people mainly focused on complex diseases such as Type-2 diabetes, obesity, etc.^13^,^14^,^15^. However, NGS-based mutation screening in IRD cohorts is still missing. Thus, it is intriguing for us to understand the genetic etiology of IRD patients in this specific ethnic group.

Here, by NGS-based target capture sequencing, we performed molecular diagnosis in a cohort of 12 Uyghur IRD probands. This approach successfully solved six IRD cases with high confidence, achieving a 50% solving rate. We also identified disease-causing alleles that represent the migration history of Uyghurs and novel disease-associated variants, which may be ethnicity-specific.

## Results

### Twelve IRD probands were collected

A total of 12 well-characterized IRD probands of Uyghur ethnicity from Moyu County, Xinjiang, China were collected in this study. Of the 12 probands, nine are diagnosed with RP, two are LCA, and one is cone dystrophy. Eight out of twelve probands have multiple affected members in the same family, while the remaining four are simplex cases.

### High quality capture sequencing data were generated

**Figure 1:**
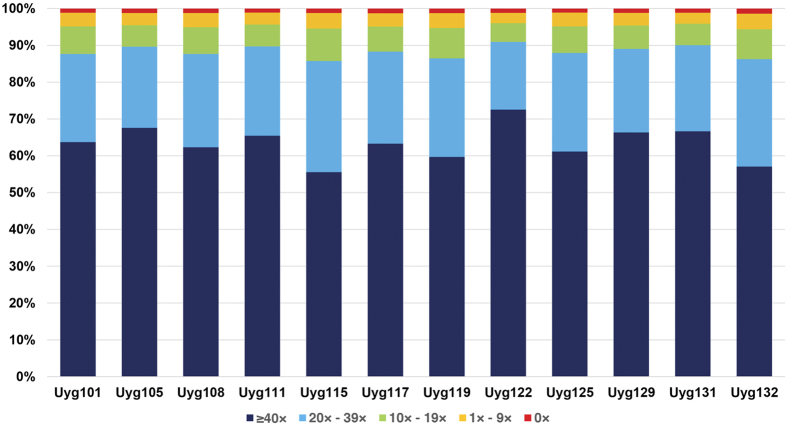
Coverage statistics on target regions in 12 IRD probands.

To identify disease-causing mutations in this IRD cohort, NGS-based target capture sequencing was performed on 12 probands. After the data acquisition and analysis, 95.2% of target regions had coverage of ≥10×, 88.3% of bases had coverage of ≥20×, and 63.4% of bases had coverage of ≥40×, indicating that sufficient sequencing coverage was achieved to enable high sensitivity for variant detection (Fig. 1). To make sure the sequencing coverage over the targeted regions was evenly distributed, evenness scores were calculated and the average is 0.8 for these 12 samples, suggesting a nearly uniform distribution of sequencing coverage.

### Pathogenic mutations were identified in 6 probands with high confidence

An average of 663 raw variants, including 605 SNPs and 58 Indels were obtained initially (Table 1). After all filtering and annotation procedures, an average of 11.2 variants (10.5 SNPs and 0.7 Indels) per sample were identified (Table 1) and were assumed as candidate pathogenic variants. By analyzing these variants, we determined known and novel plausible pathogenic mutations in 8 probands. Out of them, six probands were solved with high confidence, conferring a solving rate of 50% (6/12).

### RP cases

**Figure 2:**
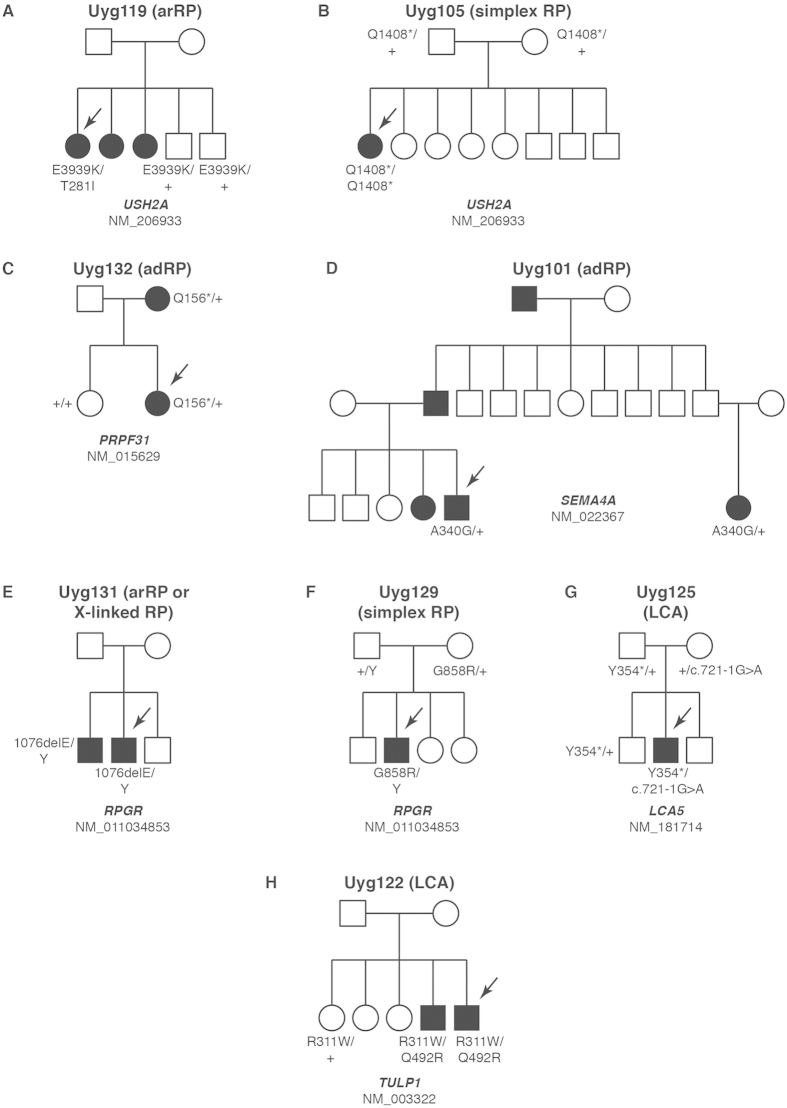
The genetic findings of the families Uyg119 (**A**), Uyg105 (**B**), Uyg132 (**C**), Uyg101 (**D**), Uyg131 (**E**), Uyg129 (**F**), Uyg125(**G**) and Uyg122 (**H**). mRNA accession ID of the disease-causing gene was labeled at the bottom of each pedigree. Genotypes (protein-altering changes based on the mRNA accession ID) were labeled next to the tested individuals. Arrows indicate the proband of each IRD family.

**Figure 3:**
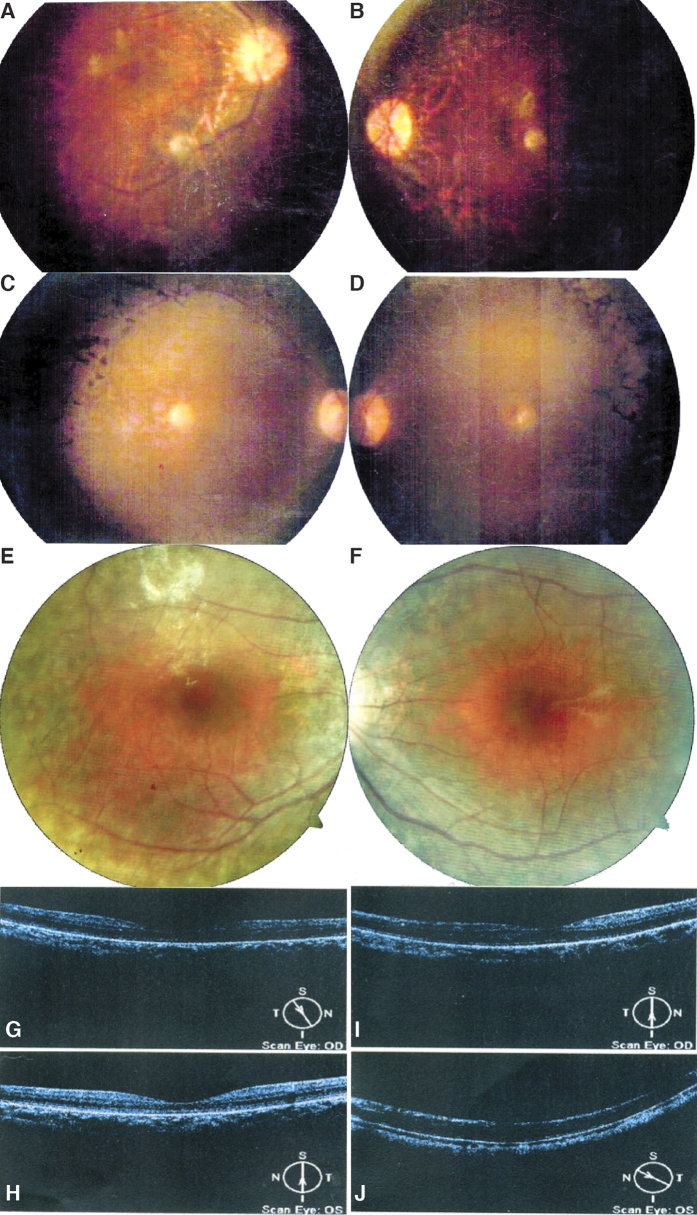
The Funduscopy images of probands Uyg119, OD (**A**) and OS (**B**); Uyg132, OD (**C**) and OS (**D**); Uyg122, OD (**E**) and OS (**F**); OCT results of the probands Uyg125, OD (**G**) and OS (**H**); Uyg122, OD (**I**) and OS (**J**).

Uyg119 is the proband of an autosomal recessive RP (arRP) family with 3 affected members in the same generation (Figs 2A and 3A,B). She is 53 years old with a visual acuity of 0.01 (decimal scale) OU. NGS data showed that she possesses biallelic missense *USH2A* variants (Table 2). One variant (c.C842 > T, p.T281I) was reported as disease-causing in patients with Usher syndrome^16^. The other variant (c.G11815 > A, p.E3939K) is novel. This novel variant is extremely rare with a population frequency of 0.0006 and is predicted to be damaging by most algorithms, suggesting *USH2A* as the disease-causing gene in this family. We then performed Sanger sequencing to confirm the variants’ identity and co-segregation with the phenotype. The results showed that two unaffected members only have one variant, indicating these two variants are *in trans*. All affected individuals in this family don’t have hearing problems.

Proband Uyg105 is a simplex RP case (Fig. 2B). She is 23 years old with a visual acuity of 0.12 OD and 0.08 OS. NGS data showed she has a homozygous stopgain variant (c.C4222 > T, p.Q1408*) in *USH2A* gene (Table 2). This variant is a known disease-causing allele for non-syndromic RP^17^. Sanger sequencing confirmed the homozygosity of this variant.

Uyg132 is the proband of an autosomal dominant RP (adRP) family with two affected individuals (Figs 2C and 3C,D). NGS identified a *PRPF31* stopgain variant (c.C466 > T, p.Q156*) (Table 2). This variant is novel but its loss-of-function nature strongly suggests the pathogenicity. Sanger sequencing showed that the proband’s affected mother also possesses this variant and her unaffected sister does not, thus confirming the co-segregation.

Proband Uyg101 is in another adRP family with incomplete penetrance (Fig. 2D). NGS identified a heterozygous variant in the adRP gene *SEMA4A* (c.C1019G, p.A340G) (Table 2). The variant is absent in control databases. It affects a highly conserved amino acid site and considered to be damaging. Sanger sequencing confirmed this variant in the proband and another distantly-related affected family member, suggesting it as the putative disease-causing variant. Since only a limited number of adRP families with *SEMA4A* variants were reported before^18^,^19^, the causality and the pathogenicity of this variant need to be validated by further experiments evaluating its functional impact.

Proband Uyg131 is in a family ascertained with arRP or X-linked RP since there are two male affected individuals in the same generation (Fig. 2E). NGS data showed that the proband possesses an inframe deletion in *RPGR* (c.3225_3227delAGA, p.1076delE) (Table 2). This variant is absent in control databases, suggesting the rareness. The putative disease-causing gene *RPGR* also agrees with the potential X-linked inheritance pattern in this family. Sanger sequencing showed that both affected individuals are hemizygous for this allele. However, since it’s an inframe deletion, the pathogenicity of this variant is still unknown and needs to be confirmed by more genetic or functional data. Therefore, the disease association of this variant was considered of low confidence.

Proband Uyg129 is a simplex RP case (Fig. 2F). NGS identified a hemizygous missense variant in *RPGR* (c.G2572 > A, p.G858R) (Table 2). The variant is not found in any control databases. The predicted protein-altering effect is “low damaging” by MutationAssessor, while it is considered as “benign” or “unknown” by other algorithms. Thus, the pathogenicity of this variant needs further validation and we categorized its disease association as low confidence.

For the remaining three RP probands, Uyg108, Uyg115 and Uyg117, there are no plausible pathogenic variants identified by our target capture sequencing, suggesting the causative allele may reside in novel disease-causing genes or deep intronic regions.

### LCA and cone dystrophy cases

Proband Uyg125 is a simplex LCA case (Figs 2G and 3G,H). NGS showed that the proband has a splicing variant (c.721-1G > A, p.?) and a deletion that creates a premature stop codon (c.1062_1068delCGAAAAC, p.Y354*) in *LCA5* (Table 2). The splicing variant is not found in any databases and the deletion was reported before as disease-causing in an LCA family^20^. Sanger sequencing showed that the splicing variant was from the proband’s mother and the deletion was from the father. The proband’s unaffected brother only possesses the deletion variant.

Proband Uyg122 is in a recessive LCA family (Figs 2H and 3E,F,I,J). He is 41 years old with a visual acuity of 0.12 OD and 0.02 OS. NGS data identified compound heterozygous variants in *TULP1*, a known LCA-causing gene (Table 2). The first variant (c.C931 > T, p.R311W) is not found in any databases and is predicted to be pathogenic by all algorithms. The second variant (c.A1475 > G, p.Q492R) is also absent in controls and is considered damaging. Sanger sequencing showed both variants exist in two affected individuals and an unaffected sister only possesses one *LCA5* variant, thus confirming the co-segregation.

Uyg111 is a 25-year-old male diagnosed with cone dystrophy. He has a visual acuity of 0.01 OU. Target capture sequencing identified no putative causative variants.

## Discussion

In this study, we performed an NGS-based molecular diagnosis on 12 IRD probands of Uyghur ethnicity, including nine RP cases, two LCA cases and one cone dystrophy case. Our method solved 6 out of 12 probands with high confidence, achieving a solving rate of 50%. In addition, we also potentially solved two cases with low confidence.

Six probands, including four RP cases (Uyg119, Uyg105, Uyg132 and Uyg101) and two LCA cases (Uyg125, Uyg122) are considered to be solved with high confidence. Specifically, in the family of Uyg119, an *USH2A* variant previously reported to cause Usher syndrome^17^ was identified in our non-syndromic RP case. A plausible explanation for the genotype-phenotype correlation is that: the other *USH2A* variant (c.G11815 > A, p.E3939K) has a milder damaging effect, thus alleviating the overall impact on the phenotype. Consistent with this explanation, the novel *USH2A* variant is not considered damaging by all prediction algorithms, suggesting its hypomorphic nature instead of complete loss-of-function. Another explanation would be that the genetic background may modify the phenotype of patients.

Two RP probands (Uyg131 and Uyg129) are solved with low confidence. For these two cases, it is unknown if the *RPGR* variants confer pathogenicity given the unsureness of predicted damaging effect. Future functional data on RPGR mutant proteins are needed to clarify this question. Nevertheless, the rareness of these two variants supports the potential pathogenicity in a human genetics perspective.

Four of our probands do not have putative causative variants identified. The missing heritability could be from disease-causing mutations in a novel disease gene. Another possibility is that the disease-causing mutations lie in the non-coding regions of known disease genes and they cannot be detected by current NGS-based target capture sequencing. Future studies by whole exome sequencing or enhanced NGS approaches that target non-coding regions would help to understand the genetic basis of current unsolved cases.

All of the patients in this study are Uyghurs from Moyu County, a town located at the south rim of Tarim Basin where the residents are almost homogeneous of Uyghur origin. Uyghurs are the descendants of Turkic-speaking people who migrated to the Tarim Basin and several other ethnicities^21^. Contemporary studies suggest that they possess a distinct genetic admixture between Europeans and East Asians and have evolved for a long time^12^,^22^,^23^. In this study, we totally identified three known disease-causing variants (Table 2). Interestingly, one known *USH2A* mutation (c.C842T, p.T281I) was reported in a patient of Turkish origin^16^, supporting the ancient migration history. One *LCA5* mutation we identified (c.1062_1068delCGAAAAC, p.Y354*), was previously reported in an Arabic Muslim family^20^, also suggesting the potential genetic influences from the Middle East. As for the third reported mutation (*USH2A*, c.C4222T, p.Q1408*), the patient ethnicity in previous literature was unknown^17^. We also identified several novel putative disease-causing variants, probably representing ethnicity-specific alleles. Further studies on larger cohorts might reveal more founder mutations that specifically exist in this ethnic group or Central Asia population. In addition, the cross-validation of the novel variants we identified in other cohorts could help us to infer the migration and evolution history of Uyghur ethnicity more accurately.

In summary, our approach identified the genetic cause of 50% IRD probands from an isolated ethnic group. A total of eight novel variants with potential pathogenicity were identified. Our study has revealed the molecular basis of IRD in Uyghurs and identified evidence of their genetic origin and migration history. Future NGS-based studies in more cohorts from this or adjacent regions will be of both scientific and cultural significance to improve our understanding of IRD molecular mechanism as well as genetic history of diverse ethnicities.

## Methods

### Clinical diagnosis of retinal disease patients

Uyghur IRD patients and other family members were ascertained at Minguang Ophthalmic Hospital (MOH), Hotan, Xinjiang, China. All patients provided written consent in accordance with the tenets of the Declaration of Helsinki. Comprehensive ophthalmic examinations including visual acuity testing (tumbling E chart), visual field testing (APS-6000, Xinchangzheng, Nanchang, China), optical coherence topography (OCT, 3D OCT-2000 Spectral Domain; Topcon, Tokyo, Japan) and funduscopy (APS-CER, Kanghua, Chongqing, China) were performed on each patient. Pedigrees were established by interviews. Genomic DNA was extracted from peripheral blood by using Qiagen kit (Qiagen Inc). All experimental methods were approved by the Institutional Review Boards of MOH and Chinese Academy of Sciences and were performed in accordance with relative guidelines and regulations.

### Capture panel sequencing

We used customized target capture for NGS. The detailed information of the capture panel can be found in previous literature^10^. Briefly, 1 μg of patients’ genomic DNA was sheared into 300–500 bp fragments. After they were end-repaired, a single adenine base was added to 3′ ends. Then we applied ligation by using adapters before DNA fragments amplification. The DNA libraries were quantified using pico green assay kit (Invitrogen, Carlsbad, CA, USA). We then washed and recovered captured DNA by Agilent Hybridization and Wash Kit. Sequencing was performed on Illumina Hiseq 2000 (Illumina, San Diego, CA, USA).

### Bioinformatics analysis

100 bp paired-end sequencing reads were obtained. Reads were aligned using BWA version 0.6.1^24^. The Genome Analysis Tool Kit version 1.05974 was used for base quality recalibration and local realignment. Atlas-SNP2 and Atlas-Indel2 were used for calling SNPs and Indels. We obtained the variant frequencies from a series of public and internal control databases including Exome Aggregation Consortium (ExAC) database, CHARGE consortium^25^, ESP-6500^26^ and 1000 Genome Project^27^. Since IRD are rare Mendelian disorders, variants with a frequency higher than 1/200 (for a recessive model) or 1/10,000 (for a dominant model) were filtered out. Then we excluded synonymous and deep intronic (distance >10 bp from exon-intron junctions) variants from further analysis. ANNOVAR (version 11/12/2014) and dbNSFP suite (version 2.9, contains SIFT, PolyPhen-2, LRT, MutationTaster, MutationAssessor, FATHMM, VEST3, etc.) were used to annotate and predict protein-altering changes. Known retinal disease-causing alleles were detected based on the HGMD professional database (version 11/15/2014).

### Sanger validation and co-segregation test

Each putative disease-causing mutation was validated by Sanger sequencing. Primer3 was used to design a pair of primers to ensure that the amplicons cover 500 bp region around the mutation site. The PCR amplicons were Sanger sequenced on an ABI 3730XL Genetic Analyzer. The results were analyzed by Sequencher 5.0.

## Additional Information

**How to cite this article**: Tajiguli, A. *et al*. Next-generation sequencing-based molecular diagnosis of 12 inherited retinal disease probands of Uyghur ethnicity. *Sci. Rep.* **6**, 21384; doi: 10.1038/srep21384 (2016).

## Figures and Tables

**Table 1:** Statistics of variants before and after filtering in 12 IRD probands.

Probands	Unfiltered variants		Inheritance model used in filtering	Variants after filtering				
	SNPs	Indels		frameshift	Inframe deletion	stopgain	splicing	missense
Uyg101	630	46	D	0	1	0	0	4
Uyg105	613	61	R	1	0	1	0	9
Uyg108	576	61	R	1	0	0	1	14
Uyg111	658	68	R	1	0	0	0	12
Uyg115	622	42	R	0	1	0	0	11
Uyg117	551	60	R	0	0	0	1	10
Uyg119	588	70	R	0	0	0	0	15
Uyg122	644	67	R	0	1	0	1	14
Uyg125	526	41	R	0	0	1	1	9
Uyg129	564	41	R	0	0	0	0	8
Uyg131	753	54	R	0	1	0	0	9
Uyg132	535	85	D	0	0	1	0	4

D, dominant (frequency cut-off: 1/10,000); R, recessive, (frequency cut-off: 1/200).

**Table 2:** The list of putative disease-causing variants identified in IRD probands.

Proband	Primary diagnosis	Gene	Accession ID	cDNA and protein changes	Genotype	Previously reported?
Uyg119	arRP	*USH2A*	NM_206933	c.G11815A, p.E3939K	Het	No
		*USH2A*	NM_206933	c.C842T, p.T281I	Het	Yes^16^
Uyg105	Simplex RP	*USH2A*	NM_206933	c.C4222T, p.Q1408*	Homo	Yes^17^
Uyg132	adRP	*PRPF31*	NM_015629	c.C466T, p.Q156*	Het	No
Uyg101	adRP	*SEMA4A*	NM_022367	c.C1019G, p.A340G	Het	No
Uyg131	arRP or X-linked RP	*RPGR*	NM_001034853	c.3225_3227delAGA, p.1076delE	Hemi	No
Uyg129	Simplex RP	*RPGR*	NM_001034853	c.G2572A, p.G858R	Hemi	No
Uyg125	LCA	*LCA5*	NM_181714	c.1062_1068delCGAAAAC, p.Y354*	Het	Yes^20^
		*LCA5*	NM_181714	c.721-1G>A, p.?	Het	No
Uyg122	LCA	*TULP1*	NM_003322	c.C931T, p.R311W	Het	No
		*TULP1*	NM_003322	c.A1475G, p.Q492R	Het	No

Het, heterozygous; Homo, homozygous; Hemi, hemizygous.
